# Long-term impact of resilience and extraversion on psychological distress during the COVID-19 pandemic: a longitudinal investigation among individuals with and without mental health disorders

**DOI:** 10.3389/fpsyt.2024.1304491

**Published:** 2024-02-15

**Authors:** Anna Schmit, Timo Schurr, Beatrice Frajo-Apor, Silvia Pardeller, Barbara Plattner, Franziska Tutzer, Andreas Conca, Martin Fronthaler, Christian Haring, Bernhard Holzner, Markus Huber, Josef Marksteiner, Carl Miller, Verena Perwanger, Roger Pycha, Martin Schmidt, Barbara Sperner-Unterweger, Alex Hofer

**Affiliations:** ^1^ Department of Psychiatry, Psychotherapy, Psychosomatics and Medical Psychology, Division of Psychiatry I, Medical University Innsbruck, Innsbruck, Austria; ^2^ Sanitary Agency of South Tyrol, Department of Psychiatry, General Hospital of Bolzano, Bolzano, Italy; ^3^ Sanitary Agency of South Tyrol, Therapy Center Bad Bachgart, Rodengo, Italy; ^4^ Department of Psychiatry and Psychotherapy B, State Hospital Hall in Tyrol, Hall in Tyrol, Austria; ^5^ Sanitary Agency of South Tyrol, Department of Psychiatry, General Hospital of Brunico, Brunico, Italy; ^6^ Department of Psychiatry and Psychotherapy A, State Hospital Hall in Tyrol, Hall in Tyrol, Austria; ^7^ Department of Psychiatry, County Hospital Kufstein, Kufstein, Austria; ^8^ Sanitary Agency of South Tyrol, Department of Psychiatry, General Hospital of Merano, Merano, Italy; ^9^ Sanitary Agency of South Tyrol, Department of Psychiatry, General Hospital of Bressanone, Bressanone, Italy; ^10^ Department of Psychiatry, County Hospital Lienz, Lienz, Austria; ^11^ Department of Psychiatry, Psychotherapy, Psychosomatics and Medical Psychology, Division of Psychiatry II, Medical University Innsbruck, Innsbruck, Austria

**Keywords:** COVID-19, mental health disorders, psychological distress, resilience, extraversion, general population, Austria, Italy

## Abstract

**Background:**

Over the past years, the COVID-19 pandemic has caused significant disruptions in daily routines. Although the pandemic has affected almost everyone, it has been particularly challenging for people with pre-existing mental health conditions. Therefore, this study investigated the long-term impact of resilience and extraversion on psychological distress in individuals diagnosed with mental health disorders (MHD) compared to the general population. In addition, possible gender-specific differences were investigated.

**Methods:**

123 patients with pre-existing MHD and 343 control subjects from Austria and Italy participated in three online surveys that had been conducted after the initial wave of the COVID-19 pandemic (t0), during the second lockdown in both countries (t1), and one year thereafter (t2). Participants completed standardized questionnaires on psychological distress (Brief-Symptom-Checklist), resilience (Resilience Scale), and extraversion (Big Five Inventory). A mediation model was employed to test the primary hypothesis. Possible gender-specific differences were analyzed using a moderated mediation model.

**Results:**

The prevalence of psychological distress was consistently higher in patients compared to controls (t0: 37.3% *vs*. 13.2%, t1: 38.2% *vs* 11.7%, t2: 37.4% *vs*. 13.1%). This between-group difference in psychological distress at the first follow-up was fully mediated by baseline resilience scores (65.4% of the total effect). During the second-follow up, extraversion accounted for 18% of the total effect, whereas resilience slightly decreased to 56% of the total effect. Gender was not a significant moderator in the model.

**Conclusion:**

Next to showing that people with MHD were particularly affected by the pandemic, these findings indicate that higher degrees of resilience and extraversion are related to less long-term psychological distress. Our findings stress the relevance of strengthening resilience and extraversion and to provide mental health support in times of crises, both to patients with MHD and the general population.

## Introduction

Since its outbreak in 2019, the Coronavirus disease 2019 (COVID-19) pandemic had a strong hold on all aspects of daily life such as healthcare, education, politics, and economy and also demonstrated the limitations of the existing healthcare systems worldwide including many fatalities. Moreover, it has placed an unprecedented burden on mental health services by affecting people of all ages and social classes and especially those with pre-existing mental health disorders (MHD) (1).

A number of globally conducted studies investigated the impact of the pandemic on mental health of the general population and the repercussions of implementing measures to contain its spread (2). However, studies focusing on its effect on individuals already struggling with MHD are scarce (3–6). Notably, even before the pandemic they were required to deal with multiple challenges, including unfair treatment, negative attitudes, and biased behavior from society (7). These stigmata, both from individuals and from society as a whole, can lead to lower rates of seeking help, less favorable outcomes in treatment, feelings of loneliness, and unstable emotions (8). On the other hand, resilience, i.e. “the capacity of a dynamic system to withstand or recover from significant challenges that threaten its stability, viability, or development” (9), may be a significant factor in protecting and maintaining mental health of any person (10, 11) and accordingly, highly resilient individuals tend to experience less psychological distress (12).

Earlier studies, both those conducted prior to (13, 14) and following the onset of the pandemic (15), have suggested that people with preexisting mental health conditions tend to exhibit lower levels of resilience. In addition to confirmation of these findings, one of our recent studies revealed that at the early stages of the pandemic, the prevalence of clinically relevant psychological distress was particularly high among those living with severe MHD and that psychological distress was predicted by a lower degree of resilience (3). Notably, resilience is partly hereditary and is composed of various multidimensional factors that interact with each other, including cognitive processes, personality traits, and constructive coping strategies (16). Focusing on improving one of these factors can therefore have a constructive effect on the others (17) and is e.g. part of cognitive behavioral therapy (18, 19). Personality traits are unique characteristics exhibited by individuals through consistent patterns of behavior and emotions (20). They have a stable component across the lifespan (21). Extraversion, for example, characterizes outgoing and optimistic individuals who tend to utilize social support more often (22) and has been associated with positive emotions (23), higher life satisfaction (24) and well-being (25, 26), less stressor-related negative affect (27), as well as with recovery (28) and growth following trauma (29). Based on this, some studies hypothesized that extraversion could positively impact psychological distress levels (30–34). Numerous studies conducted before and after the onset of the pandemic have shown that women tend to experience more psychological distress (35–40) and to have greater fears and negative expectations about the health consequences of COVID-19 compared to men (41). While males tend to have a more active coping style, females are more likely to rely on social support to reduce psychological distress (36) and are more prone to using dysfunctional coping strategies (42). Further, they tend to ruminate more on problems (43), which can prolong depressive episodes (44). Alternatively, it has been proposed that the current understanding of resilience may not fully acknowledge the intricate interplay between gender norms, societal beliefs, environmental conditions, and subjective viewpoints that contribute to women’s tendency to display lower levels of resilience when compared to men (35). On the other hand, sex differences in extraversion seem to be small with women typically scoring higher (45).

The main objective of the present longitudinal study was to investigate the mental health of residents of Tyrol (Austria) and South Tyrol (Italy) in the course of the pandemic. This report focuses on the long-term impact of resilience and extraversion on psychological distress among people diagnosed with MHD compared to participants from the general population. We hypothesized that patients would exhibit lower baseline levels of resilience and extraversion and higher levels of psychological distress during the course of the COVID-19 pandemic and that differences in psychological distress between patients and controls are partly or fully accounted for by the baseline levels of resilience and extraversion. Lastly, we were interested whether gender had an impact on the presumed associations.

## Materials and methods

### Participants

A total of 5,517 adults (≥ 18 years) who had been inpatients in a psychiatric ward in either Tyrol (N=3,928) or South Tyrol (N=1,589) in 2019 were invited by written communication to take part in an online survey, regardless of diagnosis. Among them, 123 individuals participated at all three survey time points (baseline, first follow-up t1, second follow-up t2). Diagnoses according to ICD-10 criteria were identified through the internal documentation software of participating sites.

The control group consisted of general population residents from Tyrol and South Tyrol aged ≥ 18 years who were invited to take part in this online survey through various advertising methods (e.g. flyers, social media). Participants in the control group were inquired about any diagnosed mental health disorders at study inclusion. Additionally, they were asked about an ongoing outpatient and complementary psychiatric mental health services (e.g. psychotherapy, psychological or psychiatric treatment) during each survey. If any of these questions received a positive response, those participants were excluded from the control group, and their data were omitted from the overall statistical analysis. Only those who participated in all three surveys were included in the control group. As a result, 343 individuals were assigned to the control group. After completing the survey, respondents were asked to provide their email address for follow-up surveys. As the survey took place in Austria and Italy, both the patient and the control group were required to have a good understanding of the German or Italian language.

The surveys in Tyrol and South Tyrol were conducted separately for organizational reasons, resulting in different survey periods. In Tyrol, the baseline survey took place from June 26th to September 13th, 2020, and the first follow-up survey was performed between November 30th, 2020 and January 24th, 2021. In South Tyrol, in turn, the baseline survey was conducted between September 7th and November 22nd, 2020, while the first follow-up survey was conducted from February 8th to April 4th, 2021. The time interval between surveys was the same for both regions (11 weeks). The second follow-up was conducted simultaneously in both countries (January 10th - February 21th, 2022).

It should be noted that the population of Tyrol and South Tyrol share many commonalities, making them quite comparable in various ways such as their socioeconomic conditions, healthcare systems, and other factors (46).

### Questionnaires

We conducted a longitudinal online study using the Computer-based Health Evaluation System (CHES) (47), a web-based software program. After obtaining informed consent online, we gathered sociodemographic information and COVID-19-related data. Standardized questionnaires were used to assess psychological distress, resilience, and extraversion. During the baseline survey, questions pertained to the lockdown period, while in the follow-up surveys, questions related to two weeks preceding participation.

### Sociodemographic and COVID-19-related data

The study collected sociodemographic data by inquiring about gender, age, residence, relationship status, education, current work situation and living arrangements, and severe physical health problems. Patients were also surveyed in regards of the onset of psychiatric illness and the type of currently chosen treatment.

### Psychological distress

The Brief-Symptom-Checklist is a reliable self-evaluation tool that has been used in the German (48) and the Italian version (49). It comprises 53 items and is rated using a 5-point scale (0 = not at all, 4 = extremely) to evaluate nine symptom dimensions: hostility, anxiety, depression, paranoid ideation, phobic anxiety, psychoticism, somatization, interpersonal sensitivity, and obsession-compulsion. The global severity index (GSI) is a composite score that has been used to assess psychological symptoms with a GSI T score ≥ 63 indicating clinically significant psychological distress according to Franke et al. (48). The GSI T score has been found to have an outstanding degree of external consistency (Cronbach’s α = 0.96) (50).

### Resilience

The Resilience Scale (RS-13) (51), a shortened version of the RS-25 (52) with good internal consistency (Cronbach’s α = 0.90) (51), was used to measure resilience. Respondents rated 13 items on a 7-point scale (1 = strongly disagree, 7 = strongly agree). Higher scores indicate higher resilience. According to the authors (51), scores up to 66 are categorized as low resilience, scores between 67 and 72 reflect moderate resilience, and scores of 73 or higher indicate high resilience.

### Extraversion

To assess extraversion, the extraversion subscale from the Big Five Inventory (BFI) (53) was utilized. This subscale is comprised of 8 items which are rated on a 5-point Likert scale (1 = strongly disagree, 5 = strongly agree). It demonstrates a good internal consistency with a Cronbach's α =90 (54). Possible scores range from 8 to 40 with a higher score indicating a higher level of extraversion.

### Statistical methods

For statistical analysis, IMB SPSS (version 27) was used. The statistical significance level was set to α = 5%. Metric variables were checked for deviations from normal distribution by examining kurtosis and skewness as well as visual inspection of plots. For the comparison of categorical or dichotomous sociodemographic and outcome variables between patients and control subjects Chi² tests were applied. Non-normally distributed metric variables were compared by means of Mann-Whitney U-tests. For longitudinal comparisons between baseline (t0) and follow-up (t1/t2), dichotomous variables were analyzed by Cochrane’s Q-tests, and metric variables by Wilcoxon signed rank tests.

Spearman rank correlation was used in order to detect possible associations between age, extraversion, resilience, and psychological distress at baseline and follow-ups within the patient and the control group. Fisher’s z-transformed correlation coefficients were calculated and compared between both groups. Effect sizes can be interpreted as follows: ρ, r= 0.10 - 0.29 small; ρ, r= 0.30 - 0.49 moderate; and ρ, r ≥ 0.50 high (55). An odds ratio (OR) of 1.0 indicates that there is no difference between groups. Increased odds for the patient group are present when the odds ratio is >1.0, whereas an odds ratio <1.0 indicates decreased odds for the patient group.

The main analysis consisted of mediation and moderated mediation modeling. Initially, possible (multivariate) outliers were identified by calculating leverage values, Mahalanobis distance, and Cook’s distance. Thereafter, we applied the PROCESS v4.0 macro (56) in order to fit the models and estimate the path coefficients for total, direct, and indirect effects by means of ordinary least square regressions. We calculated heteroscedasticity-consistent standard errors (HC3) (57).

## Results

### Sociodemographic characteristics

**Table 1** presents sociodemographic characteristics of the whole sample, whereas **Table 2** shows further baseline and follow-up characteristics of patients. A total of 123 patients and 343 individuals from the general population participated in the baseline survey as well as in the first and second follow-up. Patients diagnosed with disorders of adult personality and behavior (ICD-10: F60-F69) or with behavioral syndromes associated with physiological disturbances and physical factors (ICD-10: F50-F59) were not included in the analyses due to unevenly distributed psychological distress (GSI) values. This was confirmed by a statistically significant (p < 0.001) Kruskal-Wallis test and Bonferroni corrected pairwise comparison results. Hence, the patient sample was reduced by 18.7% (n=26).

**Table 1:** Baseline demographics of patients and control subjects.

Variable	Patients with MHD (N=123)	Control subjects (N=343)	Statistics^a^	p-value
	Mean ± SD or N (%)	Mean ± SD or N (%)		
Sex				
Male	54/123 (43.9%)	101/343 (29.4%)	χ²=8.52	0.004
Female	69/123 (56.1%)	242/343 (70.6%)		
Age (Years)	49.8 ± 13.5 (21-82)	45.4 ± 13.6 (18-96)	Z=-3.47	<0.001
Education (Years)	13.4 ± 4.6	15.8 ± 3.8	Z=6.03	<0.001
Residence				
Tyrol (Austria)	85/123 (69.1%)	198/343 (57.7%)	χ²=4.92	0.027
South Tyrol (Italy)	38/123 (30.9%)	145/343 (42.3%)		
Relationship				
Single	44/123 (35.8%)	65/343 (19.0%)	χ²=14.69	<0.001
Fixed partnership	78/123 (63.4%)	278/343 (81.0%)		
Work situation				
Full-time	24/123 (19.5%)	167/343 (48.7%)	χ²=31.86	<0.001
Part-time	12/123 (9.7%)	84/343 (24.5%)	χ²=12.02	<0.001
Self-employed	5/123 (4.1%)	20/343 (5.8%)	χ²=0.56	0.456
Education/training	1/123 (0.8%)	16/343 (4.7%)	χ²=3.87	0.049^b^
From home	1/123 (0.8%)	2/343 (0.6%)	χ²=0.07	0.790
Short-time work	1/123 (0.8%)	3/343 (0.9%)	χ²=0.01	0.944
Sick leave	23/123 (18.7%)	1/343 (0.3%)	χ²=62.80	<0.001
Unemployed	9/123 (7.3%)	1/343 (0.3%)	χ²=21.28	<0.001
due to COVID-19	4/123 (3.3%)	1/343 (0.3%)	χ²=7.48	0.006^b^
Retired	32/123 (26.0%)	35/343 (10.2%)	χ²=18.39	<0.001
Homemaker	5/123 (4.1%)	4/343 (1.2%)	χ²=4.02	0.045^b^
Others	5/123 (4.1%)	5/343 (1.5%)	χ²=2.93	0.087
Severe physical health problems (e.g. diabetes, cancer, etc.)	21/123 (17.1%)	25/343 (7.3%)	χ²=9.74	0.002

a Always one degree of freedom (df) unless stated otherwise. Analysis by Chi-square test (χ²), or Mann–Whitney U-test (Z).

b After Bonferroni correction (n=12) within the variable “work situation” statistical significance could not be retained.

Abbreviations: MHD = mental health disorder, SD = stndard deviation.

**Table 2:** Baseline (t0) and follow-up (t1/t2) characteristics of patients.

Variable	Mean ± SD		or N (%)	Statistics^a^	p-value
	*Median [IQR]*				
Average years since initial diagnosis of psychiatric disorder (base 2020)	11.3 ± 11.8 *6.0 [2 - 18.5]*				
Average years since first inpatient treatment due to psychiatric disorder (base 2020)	7.8 ± 9.9 *3.0 [1 – 10.5]*				
Number of patients with ICD-10 F0x.x as primary diagnosis	2/123 (1.6%)				
Number of patients with ICD-10 F1x.x as primary diagnosis	25/123 (20.3%)				
Number of patients with ICD-10 F2x.x as primary diagnosis	9/123 (7.3%)				
Number of patients with ICD-10 F3x.x as primary diagnosis	62/123 (50.4%)				
Number of patients with ICD-10 F4x.x as primary diagnosis	25/123 (20.3%)				
Current treatment due to psychiatric disorder	t0 t1 t2	89/123 (72.4%) 83/123 (67.5%) 77/123 (62.6%)↓		χ²=7.45	0.024
Psychological/psychotherapeutic treatment	t0 t1 t2	59/89 (66.3%) 53/83 (63.9%) 49/77 (63.6%)		χ²=0.11	0.949
Resident psychiatrist	t0 t1 t2	56/89 (62.9%) 49/83 (59.0%) 50/77 (64.9%)		χ²=1.52	0.467
General practitioner	t0 t1 t2	21/89 (23.6%) 18/83 (21.7%) 20/77 (26.0%)		χ²=1.63	0.444
Psychiatric outpatient clinic	t0 t1 t2	31/89 (34.8%) 27/83 (32.5%) 24/77 (31.2%)		χ²=0.18	0.913
Care facility (work)	t0 t1 t2	8/89 (9.0%) 6/83 (7.2%) 6/77 (7.8%)		χ²=1.14	0.565
Care facility (living)	t0 t1 t2	2/89 (1.1%) 2/83 (2.4%) 2/77 (2.6%)		χ²=2.00	0.368

a Cochrane’s Q-Test; always two degrees of freedom (df) unless stated otherwise.

↓ = adjusted for multiple comparisons; statistically significant (p<0.05) decrease between baseline (t0) and follow-up (t2) according to post-hoc Dunn procedure.

ICD-10 F0x.x: Organic, including symptomatic, mental disorders; ICD-10 F1x.x: Mental and behavioral disorders due to psychoactive substance use; ICD-10 F2x.x: Schizophrenia, schizotypal, and delusional disorders; ICD-10 F3x.x: Mood (affective) disorders; ICD-10 F4x.x: Neurotic, stress-related and somatoform disorders.Abbreviations: IQR = interquartile range, SD = standard deviation.

In the patient group, 56.1% were female and 69.1% lived in Tyrol, Austria, whereas in the control group, 70.6% were female and 57.7% lived in Tyrol. On average, patients were slightly older and had less years of education compared to the reference subjects. At the baseline survey, 72.4% of patients reported receiving current treatment for their MHD with the percentage decreasing to 67.5% at the first follow-up and significantly dropping to 62.6% at the second follow-up.

Half of the patient sample (50.4%) had a primary diagnosis of a mood disorder (ICD-10: F30-F39). The second most common primary diagnoses were mental and behavioral disorders due to psychoactive substance use (ICD-10: F10-F19), and neurotic, stress-related, and somatoform disorders (ICD-10: F40-F49), which had been diagnosed in 20.3% of patients, each. Over the course of three survey periods, patients most frequently reported psychological/psychotherapeutic treatment and visits to resident psychiatrists as their preferred treatment options.

### Differences in psychological distress, resilience, and extraversion between patients and control subjects

Over the course of time, the level of psychological distress remained relatively stable among both patients and control subjects. However, individuals pertaining to the control group constantly achieved significantly lower psychological distress scores compared to patients. The odds for patients reporting a GSI T-value ≥ 63 (clinically relevant psychological distress) were 392% (t0), 469% (t1), and 396% (t2) higher compared to controls.

At baseline, patients achieved significantly lower resilience scores compared to controls with a mean RS-13 score of 61.2 ± 15.7 versus 73.9 ± 9.2. The majority (61.0%) of patients achieved low RS-13 scores (≤66), while 61.2% of the controls achieved high RS-13 scores (≥73). Similarly, patients reported lower levels of extraversion than controls with a mean score of 24.8 ± 6.0 versus 29.0 ± 5.4. Details are shown in **Table 3**.

**Table 3:** Construct characteristics of baseline (t0) and follow-up (t1/t2) questionnaire characteristics in patients and control subjects.

Construct/Questionnaire		Patients with MHD (N=123)	Control subjects (N=343)	Statistics^a^	p-value
		Mean ± SD *Median [IQR]*	Mean ± SD *Median [IQR]*		
Psychological distress (BSCL) Global Severity Index (GSI)	t0	0.83 ± 0.72 *0.64 [0.26-1.14]*	0.44 ± 0.43 *0.30 [0.13-0.55]*	Z=-5.50; r=0.25	<0.001
	t1	0.80 ± 0.63 *0.62 [0.30-1.26]*	0.44 ± 0.45 *0.32 [0.13-0.58]*	Z=-5.83; r=0.27	<0.001
	t2	0.78 ± 0.64 *0.66 [0.25-1.11]*	0.45 ± 0.43 *0.30 [0.13-0.63]*	Z=-5.12; r=0.24	<0.001
**Resilience** (RS-13; range: 13-91)	t0	61.2 ± 15.7 *62* [50–71]	73.9 ± 9.2 *75 [69-80]*	Z=8.31; r=0.38	<0.001
**Extraversion** (BFI; range: 8-40)	t0	24.8 ± 6.0 *25.0 [20.5-29.0]*	29.0 ± 5.4 *29.0 [25.0-33.0]*	Z=6.41; r=0.30	<0.001
Psychological distress (BSCL) Global Severity Index (GSI)		% (N)	% (N)		
		T value ≥63	T value ≥63		
	t0	37.3% (44/123)	13.2% (45/343)	χ²=32.74; OR=3.92	<0.001
	t1	38.2% (47/123)	11.7% (40/343)	χ²=42.03; OR=4.69	<0.001
	t2	37.4% (46/123)	13.1% (45/343)	χ²=33.96; OR=3.96	<0.001
Resilience (RS-13)					
Low (score ≤66)	t0	61.0% (75/123)	17.4% (59/343)	χ²=84.68; OR=7.52	<0.001
Moderate (score 67-72)	t0	16.3% (20/123)	21.5% (73/343)	χ²=1.43; OR=0.72	0.232
High (score ≥73)	t0	22.8% (28/123)	61.2% (208/343)	χ²=51.96; OR=0.19	<0.001

a Analysis by Chi-square test (χ²), or Mann-Whitney U-test (Z).

Abbreviations: BFI = Big Five Inventory, BSCL = Brief-Symptom-Checklist, IQR = interquartile range, MHD = mental health disorder, OR = odds ratio, RS-13 = Resilience Scale, SD = standard deviation.

### Association of resilience, extraversion, and psychological distress

As presented in **Table 4**, we detected a significant moderate to high inverse relationship between baseline resilience and psychological distress at follow-up among both groups. At t1, this association was significantly stronger in patients compared to controls. We also found a significant low to moderate inverse relationship between baseline extraversion and psychological distress at follow-up and again, this association was significantly stronger in patients compared to controls at t1. Furthermore, patients and controls demonstrated a significant moderate to high positive correlation between resilience and extraversion and a low to moderate negative correlation between age and psychological distress. The latter was consistently stronger in patients compared to controls.

**Table 4:** Spearman rank correlations of variables for mediation analysis within the patient (N=123) and the control group (N=343).

	Control subjects				Patients with MHD			
Variable	Age	Extraversion (t0)	Resilience (t0)	Psychological distress (t1)	Age	Extraversion (t0)	Resilience (t0)	Psychological distress (t1)
Extraversion (t0)	-.050				.116			
Resilience (t0)	.044	.418***			.188*	.518***		
Psychological distress (t1)	-.135*	-.249***	-.448***		-.341***	-.458***	-.608***	
Psychological distress (t2)	-.097	-.295***	-.418***	.784***	-.299***	-.387***	-.504***	.821***

* p <0.05; *** p <0.001.

Fisher’s z transformed testing of correlation coefficients differed statistically significant between both groups (patients vs. control subjects) regarding the following comparisons (after Bonferroni correction statistical significance was not retained): Age vs. psychological distress (t1) (z=-2.07, p=0.039); extraversion vs. psychological distress (t1) (z=-2.26, p=0.024); resilience vs. psychological distress (t1) (z=-2.11, p=0.035); age vs. psychological distress (t2) (z=-1.99, p=0.047).

Abbreviation: MHD = mental health disorders.

### Mediation analyses

#### Baseline resilience and extraversion as mediators of the relation between patients/control subjects and psychological distress at follow-up

According to the results of analysis for (multivariate) outliers using Cooks distance, leverage values, and Mahalanobis distance, previously set limits were not exceeded. Hence, outliers were not detected. Concerning possible interaction effects between the independent grouping variable (patients/control subjects) and the mediators (resilience and extraversion), analyses did not reveal statistically significant results. Initially, we included age and place of residence (Austria/Italy) as covariates in all mediation models. However, there is the possibility of over-adjustment modelling with this approach. As a result, the model would fit the data very well, but it would be more challenging to generalize and apply for further research. We therefore compared models including age with residence and only age as covariates. The findings indicated that there was very little (<5%) change in the coefficients’ sizes of the mediation models. For this reason, we opted for the more parsimonious models with age as the only covariate.

**Figure 1:**
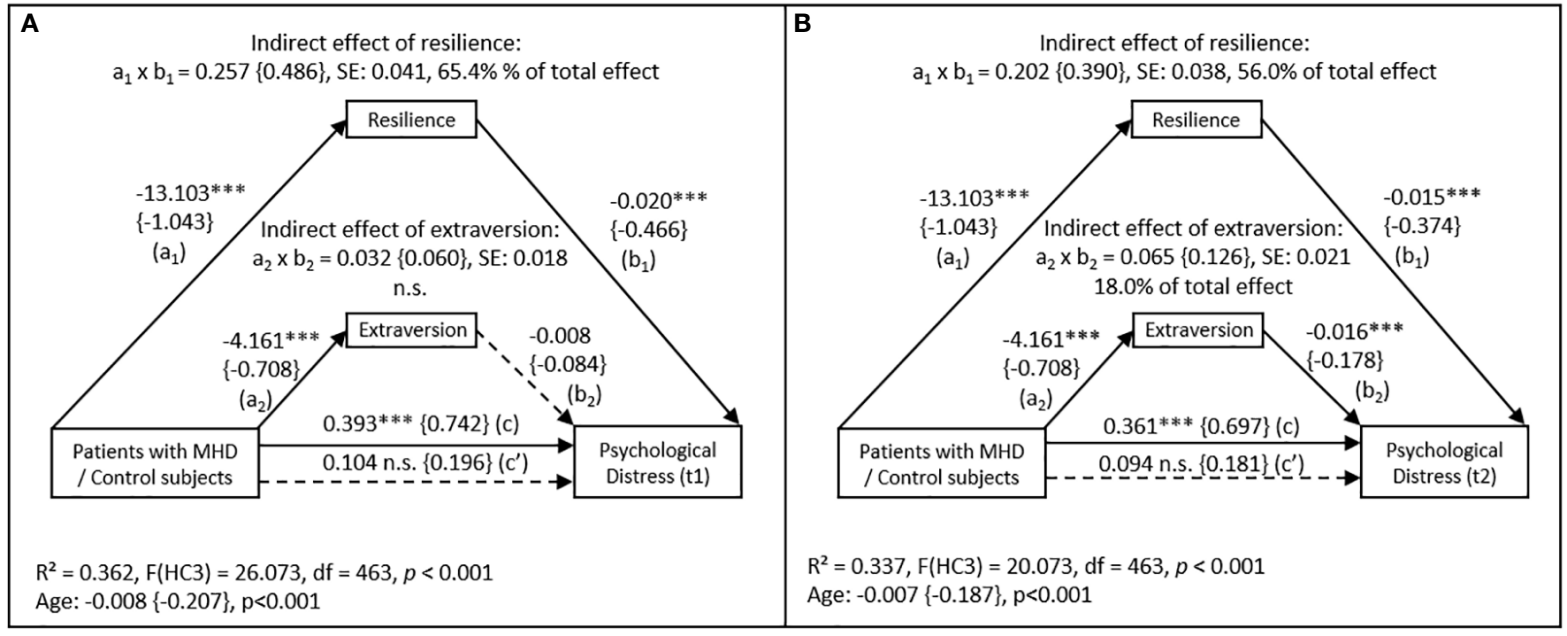
Mediation model with the effect of patients and control subjects on psychological distress (BSCL) at **(A)** first follow-up (t1) and **(B)** second follow up (t2) mediated by resilience (RS-13) and extraversion (BFI) measured at baseline (N=466). Abbreviations: n.s. = not significant; SE = standard error; MHD = mental health disorders. Control subjects are coded with (0), patients are coded with (1). Values in curved brackets represent completely standardized coefficients (β) for metric and partially standardized coefficients for dichotomous variables. Coefficient of determination (R²), coefficients and p-values of the “age” covariate included in the model are depicted for the total effect model. Heteroscedasticity-consistent standard errors (HC3) and 95% confidence intervals are based on 10.000 percentile bootstrapped samples. Solid lines indicate statistically significant connections. *** *p <*0.001.

**Figure 1A** depicts the findings for the mediation analysis of the whole sample with patients/control subjects as independent variable (X), baseline resilience and extraversion as mediators (M), and psychological distress at first follow-up as dependent variable (Y). Results show that the difference between patients and controls regarding psychological distress was almost fully attributable (65.4% of the total effect) to the mediating effect of resilience (a_1_ × b_1 _= 0.257; 95% CI [0.182-0.636]). For the indirect effect of extraversion (a_2_ × b_2_) no statistically significant results were obtained. Due to the mediation, the mean difference in psychological distress between patients and controls was reduced from 0.393 (total effect, c) to 0.104 (direct effect, c´).

**Figure 1B** shows again the mediation analysis for the whole sample. Except for the dependent variable (psychological distress at second follow-up), all variables included were the same as described above. Here, the indirect effect of resilience decreased, but remained significant (a_1_ × b_1 _= 0.202; 95% CI [0.131-0.282]), now attributable for 56.0% of the differences in psychological distress between patients and controls. However, the indirect effect of extraversion became statistically significant (a_2_ × b_2 _= 0.065; 95% CI [0.028-0.109]) with a proportion of 18.0% of the total effect.

#### Gender-specific and combined analyses: mediation and moderated mediation models

**Figure 2:**
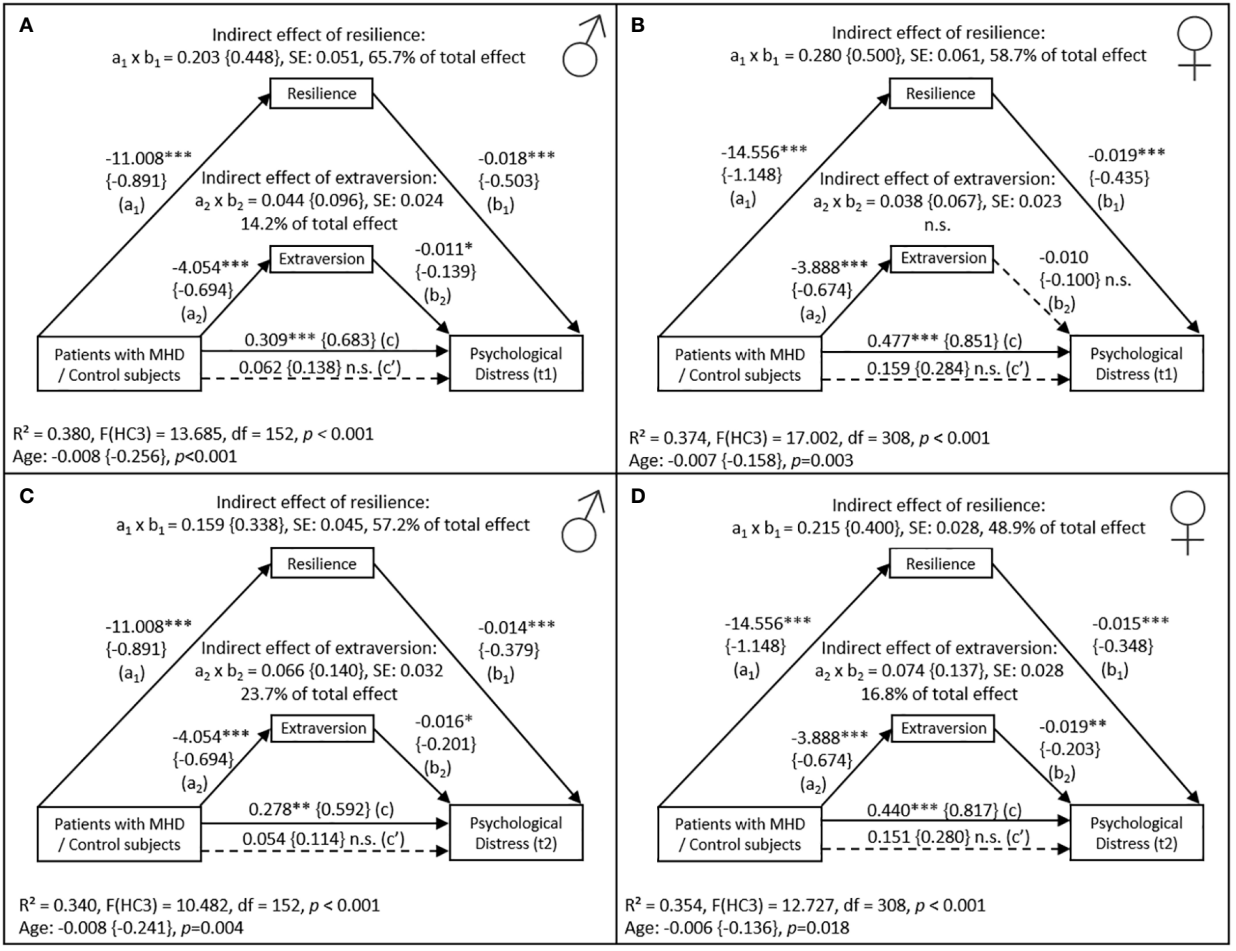
Mediation model with the effect of patients and control subjects separated by gender (N_Men_=155; N_Women_=311) on psychological distress (BSCL) at first follow-up (t1) in men **(A)** and in women **(B)**, and at second follow-up (t2) in men **(C)** and in women **(D)**, mediated by resilience (RS-13) and extraversion (BFI) measured at baseline. Abbreviations: n.s. = not significant; SE = standard error; MHD = mental health disorders. Control subjects are coded with (0), patients are coded with (1). Values in curved brackets represent completely standardized coefficients (β) for metric and partially standardized coefficients for dichotomous variables. Coefficient of determination (R²), coefficients and p-values of the “age” covariate included in the model are depicted for the total effect model. Heteroscedasticity-consistent standard errors (HC3) and 95% confidence intervals are based on 10.000 percentile bootstrapped samples. Solid lines indicate statistically significant connections. * *p <*0.05, ** *p <*0.01, *** *p <*0.001.

Initially, for the sake of simplicity of modeling, mediation analyses were performed for male and female individuals separately. **Figures 2A, B** show the mediation models separated for genders including patients/control subjects as independent variable (X), psychological distress at first follow-up as dependent variable (Y), and resilience as well as extraversion as mediators (M). The indirect effect of resilience appeared to be lower in male (a_1_ × b_1 _= 0.203 (95% CI [0.110-0.308])) compared to female subjects (a_1_ × b_1 _= 0.280 (95% CI [0.169-0.409])). Moreover, the mediating effect of extraversion was statistically significant in male (a_2_ × b_2 _= 0.044 (95% CI [0.002 – 0.097])) but not in female individuals (a_2_ × b_2 _= 0.038 (95% CI [-0.004 - 0.087])).

**Figures 2C, D** depict mediation models including independent and mediator variables similar to those described above, however, here we used psychological distress measured during the second follow-up as dependent variable (Y). In both female and male participants the mediating effect of resilience decreased, accounting for 48.9% (female) and 57.2% (male) instead of 58.7% (female) and 65.7% (male) in the differences in psychological distress between patients and controls. In contrast, the mediating effect of extraversion became significant in female (a_2_ × b_2 = _0.074 (95% CI [0.026 – 0.136])) and increased in male study participants (a_2_ × b_2 = _0.066 (95% CI [0.013-0.136])). Concerning the direct effect of resilience on psychological distress (b_1_) at second follow-up, effect sizes were lower among both males and females compared to the first follow-up (b_1,females_= -0.015, b_1,males_= -0.014).

Since female patients seemed to achieve lower mean resilience scores compared to their male counterparts (a_1, female_ = -14.556 versus a_1,male_ = -11.008) and slightly higher levels of extraversion (a_1, female_ = -3.888 versus a_1,male_ = -4.054), we tested whether gender was a moderator of the relationship between the grouping variable (patients/control subjects) and the mediators (resilience and extraversion). Furthermore, as the effect of resilience on psychological distress seemed to decrease whereas the effect of extraversion increased, we were interested whether gender was a moderator between both mediators and psychological distress (first and second follow-up). Hence, we employed two moderated mediation models. Again, variables were entered in the first model (PROCESS: model 7) as described above, including gender as a moderator of the relation between the grouping variable and the mediators. In the second model (PROCESS: model 14), we used gender as a moderator between both mediators and psychological distress. Results of both models employed indicated that the size of the indirect effects were not dependent on gender. Hence, the mediation of resilience and extraversion on the effect of the grouping variable on psychological distress was not different between males and females.

## Discussion

The present study investigated the long-term impact of resilience and extraversion on psychological distress in patients diagnosed with MHD compared to control subjects out of the general population of Tyrol and South Tyrol. Results revealed that the prevalence of clinically relevant psychological distress was almost three times higher among patients at any time of the survey and that baseline resilience mediated this between-group difference at both follow-ups, whereas the indirect effect of extraversion measured at baseline became only significant at the second follow-up. Gender was not a significant moderator in this model. However, resilience was constantly found to have a stronger indirect effect on psychological distress among females compared to males. Extraversion, in turn, only had a significant indirect effect on psychological distress among males at the first follow-up, while its mediating effect became significant among females and increased among male study participants at the second follow-up.

In the course of the pandemic, psychological distress did universally significantly increase among the general population (58, 59), including residents of Austria (60) and Italy (61). It has been shown in this context that a person’s mental strain can be influenced by various factors like the country of residence (59), the condition of local healthcare and social security systems (62), as well as by gender, marital status, and family situation (33). In the United States, 12-15% of the general population have reported symptoms of serious psychological distress over an extended period (63), which is in line with our findings. However, this prevalence was found to have been even higher in other countries. A cross-sectional survey conducted in Ethiopia at the beginning of the pandemic, for example, revealed that about one quarter of study participants perceived high to very high psychological distress (64).

We found that patients experienced significantly higher levels of psychological distress compared to the general population over time and that the prevalence of psychological distress did not significantly change from baseline to both follow-ups in both groups. These results are generally in accordance with those of other researches. Scheeren et al. (65), for example, observed consistent levels of psychological distress among both autistic and non-autistic adults, and a representative study from Austria reported that the number of study participants out of the general population with clinically relevant symptoms remained unchanged from April to September 2020 (66). Investigating another sample in Austria, Oppenauer et al. (67), in turn, found that patients with mental health conditions reported the highest levels of psychological distress, which again supports our findings.

At baseline, 72.4% of study participants who had been admitted to psychiatric facilities in Tyrol and South Tyrol in 2019 sought some form of psychiatric help. This number dropped to 67.5% at the first follow-up survey and to 62.6% at the second. Despite this decrease in the use of professional help, patients continued to report clinically relevant levels of psychological distress throughout our survey. In theory, this should had led to a higher demand for mental health services as described by the COVID-19 practitioner survey 2021 of the American Psychological Association (68). On the other hand, due to the implementation of lockdown and government restrictions, patients may have stopped seeking psychiatric help at the beginning of the pandemic. In Austria, for example, the government advised people to avoid hospitals for non-essential health problems to free up capacity for COVID-19 patients (69). As a result, the access to medical facilities reduced significantly, including outpatient care, private practices, and rehabilitation centers. Additionally, patients may have been afraid of SARS-CoV-2 infection and may thus voluntarily have waived psychiatric assistance (70). This may have led to a decrease in the number of psychiatry emergency department visits and inpatient occupancy rates as it has been observed in the largest mental health hospital in Canada (71). It is likely that there was a sudden surge in demand for mental health treatment among those with pre-existing or newly developed mental health conditions following the initial lockdown. As a consequence, people who had been managing their conditions effectively might have experienced challenges in accessing professional care from psychiatrists or psychologists. The already substantial pre-pandemic waitlist for therapy could have become even more pronounced during this time (68). These factors may have contributed to a reduced utilization of mental health services and high levels of continuing psychological distress.

Pre-pandemic research had already shown that individuals diagnosed with MHD exhibit lower resilience (14, 72) and less extroverted traits (73, 74) compared to those without MHD. Similarly, significantly lower resilience levels were observed among psychiatric patients during the COVID-19 pandemic (3, 15). This is of particular relevance as the degree of resilience is known to correlate with well-being, quality of life, as well as with the improvement in mental health and recovery (75) in patients with MHD. In the clinical setting, higher resilience shows better treatment response (76) as well as a lower prevalence (77) and a lower severity of posttraumatic stress disorder (78). As expected, we found a strong negative correlation between resilience and psychological distress among both patients and controls. In addition, baseline resilience mediated in large part the between-group difference in psychological distress at both follow-ups. Notably, the indirect effect of resilience on psychological distress was more pronounced among females compared to males, indicating that the former tend to benefit more from being resilient. This corroborates some of our previous findings (33, 79). Interestingly, the mediating effect of resilience decreased slightly from t1 to t2, indicating that participants may have adapted to the prevailing situation. To further reduce psychological distress, other mediation factors may have increased in importance, e.g. extraversion as shown in our analyses. Extraversion has previously been demonstrated to represent the personality trait with the strongest correlation to overall well-being among the big five personality traits (25). It has been associated with high resilience, positive affect, and mental health in general (80). However, research done during the pandemic has yielded conflicting results. One the one hand, high extraversion has been linked to greater stress levels (81), while another study found it to be correlated with better emotional, psychological, and social well-being (80). These studies have been carried out in Canada at the beginning of the pandemic and did not consider its long-term effects, gender disparities, or medical history. In the current study, extraversion mediated the difference in psychological distress between patients and control subjects. In the short-term (t1), this effect was significant in males only, however, in the long-term (t2), it was more pronounced in females. Although suggesting that both resilience and extraversion might have become more crucial for females than for males in the long term, moderated mediation analysis did not show significant differences between genders. We therefore assume that independently of gender, the relevance of extraversion may not be particularly important during the early stages of a stressful situation but may increase over time as the situation stabilizes and becomes the “new normal”. Although COVID-19 restrictions have been lifted in the meantime, there is still evidence of social distancing. Post-COVID-19 reality can vary largely between individuals, ranging from job loss to seeking new employment due to workforce reductions. For some it may involve grieving the loss of a loved one due to COVID-19, while for others, it may entail losing social connections and friends because of social distancing measures implemented during lockdowns. Thus, in all these new post-COVID-19 realities, extraversion may take on a significant meaning. Being extraverted is usually accompanied by an extensive circle of friends (82) and is linked to the ability to keep in touch with others and form new friendships (83). Hence, individuals who are more extraverted may nowadays experience long-term benefits in their social interactions and consequently social support, which, in turn, may lead to less psychological distress. It has to be mentioned, however, that over a period of two years after the COVID-19 outbreak, a decline in extraversion has been observed in the American population (84). Sutin et al. suggest that major stressful events can have an impact on personality, especially in younger adults (84). Given the possibility that extraversion may have decreased during the pandemic, the positive aspect of extraversion should be taken into greater consideration. It claims for the development of strategies that encourage and support more extraverted behavior as this could create more social contact and increase individual social support. Ultimately, this could increase resilience and reduce psychological distress.

Despite the implications of our findings, the current study also has some limitations. The survey was voluntary and relied on self-reported information only. Accordingly, social-desirability bias has to be taken into account next to recall bias. As this study was an online self-report survey, diagnoses of participating individuals with mental health disorders who had been treated as inpatients in 2019 were gathered from chart information and there were no standardized assessments of psychopathology at the times of the survey. We therefore cannot provide information on the presence of psychiatric diagnosis at t1 and t2. Additionally, there are limitations in the generalizability of our results. These constraints include a limited number of participants and a decreasing response rate from baseline to the second follow-up as well as different levels of COVID-19 confinement measures due to a transnational sample. Furthermore, we did not have pre-pandemic baseline measures, making it difficult to determine whether the observed psychological distress was solely due to the pandemic. Despite these limitations, the study’s longitudinal design is a notable strength. This research is also the first to investigate the mediating role of resilience and extraversion on psychological distress among the population of Tyrol and South Tyrol. In addition and notably, it suggests a long-lasting negative impact of the pandemic on mental health of people with and without MHD. It is imperative that we work towards providing more comprehensive mental health services and improving accessibility for those who are in need to overcome this and future crises.

## Data availability statement

According to the Austrian and Italian law, data sharing requires approvals from the regional Committees for Medical and Health Research Ethics and from the regional Data Protection Officers. The data are therefore not publicly available. The data that support these findings can be provided by TS, Medical University Innsbruck, upon reasonable request.

## Ethics statement

This study was reviewed and approved by the ethics committees of the Medical University Innsbruck, Austria (approval number 1147/2020) and of the Sanitary Agency of South Tyrol, Italy (approval number 83/2020) and all participants provided informed consent online.

## Author contributions

AS: Investigation, Methodology, Resources, Visualization, Writing – original draft, Writing – review & editing. TS: Data curation, Formal analysis, Methodology, Resources, Visualization, Writing – original draft, Writing – review & editing. BF-A: Conceptualization, Methodology, Project administration, Resources, Supervision, Writing – review & editing. SP: Conceptualization, Methodology, Project administration, Resources, Writing – review & editing. BP: Conceptualization, Methodology, Project administration, Resources, Supervision, Writing – review & editing. FT: Investigation, Methodology, Resources, Writing – review & editing. AC: Methodology, Resources, Writing – review & editing. MF: Methodology, Resources, Writing – review & editing. CH: Methodology, Resources, Writing – review & editing. BH: Conceptualization, Methodology, Resources, Software, Writing – review & editing. MH: Methodology, Resources, Writing – review & editing. JM: Methodology, Resources, Writing – review & editing. CM: Methodology, Resources, Writing – review & editing. VP: Methodology, Resources, Writing – review & editing. RP: Methodology, Resources, Writing – review & editing. MS: Methodology, Resources, Writing – review & editing. BS-U: Methodology, Resources, Writing – review & editing. AH: Conceptualization, Funding acquisition, Methodology, Project administration, Resources, Supervision, Validation, Visualization, Writing – review & editing.
